# Editorial: Lipid regulation in plants: a spatiotemporal approach to environmental stress adaptation

**DOI:** 10.3389/fpls.2026.1905753

**Published:** 2026-07-09

**Authors:** Yueh Cho, Ying-Chen Lin

**Affiliations:** 1 Bachelor Program of Biotechnology and Food and Nutrition, National Taiwan University, Taipei, Taiwan; 2 Institute of Biotechnology, National Taiwan University, Taipei, Taiwan; 3 Chinese Academy of Medical Sciences Oxford Institute, Nuffield Department of Medicine, University of Oxford, Oxford, United Kingdom

**Keywords:** fatty acid remodeling, lipid metabolism, lipid signaling, lipidomics, PI-PLC, plant abiotic stress, spatiotemporal dynamics, thermotolerance

## 1 Lipids as dynamic regulators of stress adaptation

In the natural environment, plants are continuously exposed to suboptimal conditions, including extreme temperatures, soil salinity, and drought. These abiotic stressors directly perturb biological membranes, often leading to downstream transcriptional responses (Hou et al., 2016). Because membranes are predominantly composed of lipids, the membrane lipidome is among the earliest cellular targets of environmental fluctuation, and its dynamic remodeling is central to stress perception and cellular protection (Hou et al., 2016).

One of the best-characterized adaptive mechanisms involves changes in membrane lipid desaturation. Under cold stress, the enrichment of membrane lipid species containing 18:2 and 18:3 fatty acid chains reflects the increased incorporation of polyunsaturated fatty acids (PUFAs), which prevents membrane rigidification (Barrero-Sicilia et al., 2017). Under heat stress, sequestration of 18:3 chains from the membrane bilayer limits susceptibility to ROS-mediated lipid peroxidation, thus maintaining overall membrane integrity during acute heat shock (Niu and Xiang, 2018; Spivey et al., 2023; Narayanan et al., 2020; Zoong Lwe et al., 2020). Therefore, modulating PUFA levels has emerged as an important strategy for improving thermotolerance, particularly in reproductive tissues that undergo rapid membrane expansion during flowering and seed development.

Under sustained or extreme abiotic stress, plants also divert membrane-derived fatty acids into triacylglycerol (TAG) in lipid droplets and plastoglobules. This sequesters potentially toxic intermediates and buffers stress-induced ROS. NAC, WRKY, MYB, and bHLH transcription factors regulate TAG accumulation (Nam et al., 2022), illustrating how transcriptional reprogramming directly shapes lipid remodeling.

In addition to their structural roles, membrane lipids participate directly in stress signaling: phosphoinositide-specific phospholipase C (PI-PLC) hydrolyzes phosphoinositides to generate diacylglycerol (DAG) and inositol phosphates, with DAG converted to phosphatidic acid (PA), a major second messenger (Laxalt et al., 2025). These signals enable rapid and spatially localized stress responses, particularly in roots exposed to environmental fluctuations.

This Research Topic examines lipid regulation in four crops that underpin global food, fiber, and bioenergy production: upland cotton (*Gossypium hirsutum*), oilseed rape (*Brassica napus*), foxtail millet (*Setaria italica*), and pennycress (*Thlaspi arvense* L.). In these systems, temperature extremes and salinity perturb membrane lipid homeostasis and compromise cellular metabolism and reproductive development. The four contributions illustrate how spatiotemporal lipid dynamics, from chromatin-mediated regulation of fatty acid biosynthesis and organ-specific signaling to developmental-stage-specific membrane remodeling, shape plant resilience under abiotic stress.

## 2 Temporal and epigenetic control of FA biosynthesis

Yang et al., addressed this critical temporal dimension through a multi-omics study on upland cotton (*G. hirsutum*), comparing a cold-tolerant line (Xinluzao 52, X52) and a cold-sensitive line (Dai 4554, D4554) at 0 h versus 6 h of cold treatment. By integrating ATAC-seq with transcriptomics and metabolomics, they elucidated a regulatory network linking chromatin state, TFs, FA biosynthetic enzymes, and FA metabolites. DEGs in X52 were specifically enriched in the FA metabolism pathway during early cold exposure, an enrichment absent in D4554. ATAC-seq identified thousands of differentially accessible chromatin regions (DARs) enriched for stress- or lipid-associated transcription factor binding motifs. Therefore, chromatin acts as a temporal gatekeeper, conditionally opening specific loci to rapidly mobilize FA biosynthetic programs and ensure that membrane lipid composition is precisely adjusted to survive acute temperature drops.

## 3 Spatial and developmental gating of membrane remodeling

Heat stress presents the opposite biophysical challenge of cold stress (Milovskaya et al., 2026; Niu and Xiang, 2018): high temperatures induce membrane hyper-fluidity and increase PUFA susceptibility to ROS-mediated lipid peroxidation, damaging the membrane. How PUFA modifications affect tissues and developmental stages has remained an open question. Jaikumar et al. addressed this Research Topic using CRISPR-edited lines of pennycress (*Thlaspi arvense* L.) mutated in *FATTY ACID DESATURASE 2* (*FAD2*) and *REDUCED OLEATE DESATURATION 1* (*ROD1*) to examine heat tolerance during flowering and silicle development. FAD2 generates linoleic acid (18:2) from oleic acid (18:1) and broadly affects membrane composition, whereas ROD1 (PDCT) predominantly affects TAG composition in oil-accumulating tissues, including pollen. At 28 °C, pollen viability reached 71% (*fad2*) and 54% (*rod1*) versus 37% in the wild type; heat-stress yield losses fell from 61% in the wild type to 40% (*rod1*) and 0% (*fad2*); *fad2* silicles also showed 65% less lipid peroxidation. Thus, reproductive thermotolerance is developmentally gated: rapid membrane expansion and TAG-derived lipids for pollen tube growth impose unique lipid requirements, and strategic PUFA reduction secures yield under thermal stress, providing an auxiliary benefit alongside improved seed-oil quality.

## 4 Spatial precision in lipid signaling networks

Beyond structural remodeling, minor membrane phospholipids act as spatially precise second messengers. PI-PLCs hydrolyze phosphoinositides to generate DAG and inositol phosphates, with DAG converted to PA, a second messenger that feeds ABA-responsive and other adaptive cascades (Laxalt et al., 2025). Zhu et al. illustrated this spatial specificity in foxtail millet (*Setaria italica*), a C4 crop noted for its broad resilience to abiotic stress. Phylogenetic analysis identified five SiPLC members. Of these, SiPLC1 was found to play a highly specialized, anatomically restricted role: predominantly expressed in roots, it is the most strongly salt-induced, and heterologous overexpression in Arabidopsis lines exhibited higher NaCl tolerance than the WT. The presence of promoter ABA-responsive motifs, the induction of *NCED3*, *RD29A*, *RD22*, and *KIN1*, and the suppression of ABI1 in overexpression lines place SiPLC1 in an ABA-dependent salt response, exemplifying how plants position PI-PLC isoforms in anatomically strategic tissues, such as roots, which are the first interface with soil salinity.

## 5 Mapping the spatiotemporal lipid landscape

Traditional bulk lipidomics obscures the spatial reality of lipid distribution across cell layers, organelles, and membrane contact sites (Jouhet et al., 2025). A review by Liao et al. on oilseed rape (*Brassica napus*) highlighted how matrix-assisted laser desorption/ionization mass spectrometry imaging (MALDI-MSI; (Boughton and Thinagaran, 2018; Horn and Chapman, 2023) maps distinct lipid species while preserving anatomical context, revealing developmental shifts in lipid biosynthesis. Integrating MSI with single-cell transcriptomics will be essential for deciphering how tissues such as the epidermis versus the mesophyll or the vegetative leaf versus the developing anther differentially remodel their lipidomes under environmental fluctuation.

## 6 Concluding remarks

The majority of molecular insight into stress-driven lipid remodeling still derives from *Arabidopsis* under controlled conditions, and translating this knowledge to crops remains a major challenge (Scholz et al., 2024). The four studies collected here extend these translational efforts across cotton, pennycress, foxtail millet, and oilseed rape, where lipid-mediated stress responses are species-specific and shaped by tissue type and developmental stage, particularly in reproductive organs that directly determine yield. From the chromatin-level temporal orchestration of fatty acid biosynthesis (cotton) through developmentally gated PUFA-dependent thermotolerance (pennycress) and spatially localized PI-PLC signaling in roots (*foxtail millet*) to the imperative of high-resolution spatial imaging (oilseed rape), these articles provide a comprehensive roadmap. Resolving the precise spatiotemporal dynamics of lipid remodeling will support targeted strategies for engineering climate-resilient crops capable of securing global food supplies under unprecedented environmental volatility.
